# Efficient green silver nanoparticles-antibiotic combinations against antibiotic-resistant bacteria

**DOI:** 10.1186/s13568-023-01619-7

**Published:** 2023-10-17

**Authors:** Muhammad Adil, Siyab Alam, Urooj Amin, Irfan Ullah, Mian Muhammad, Muti Ullah, Asma Rehman, Tariq Khan

**Affiliations:** 1 Department of Biotechnology, University of Malakand, Chakdara, 18800 Dir Lower Pakistan; 2 CAS Key Laboratory for Biomedical Effects of Nanomaterials and Nanosafety and CAS Center for Excellence in Nanoscience, National Center for Nanoscience and Technology, University of Chinese Academy of Sciences, Beijing, China; 3 Department of Chemistry, University of Malakand, Chakdara, 18800 Dir Lower Pakistan; 4 Institute of Pathology and Diagnostic Medicine, Khyber Medical University, Peshawar, Pakistan; 5 Nanobiotechnology Group, Industrial Biotechnology Division, National Institute for Biotechnology and Genetic Engineering (NIBGE), Faisalabad, Punjab Pakistan

**Keywords:** Silver nanoparticles, Cephalosporins, Antibiotics, *Mentha longifolia*, Antibiotic resistant bacteria

## Abstract

Antibiotic-resistant bacterial strains and the consequent surge in infections caused by them have become major public health concerns. Silver nanoparticles (AgNPs) exhibit antibacterial properties and have wide applications in biomedical sciences. In this study, AgNPs were synthesized in the presence of antibiotics: Ceftazidime (Cft), Cefotaxime (Cef), Ceftriaxone (Cfx), and Cefepime (Cpm), along with the extract of *Mentha longifolia*. *Mentha longifolia*-based AgNPs were kept as the control for all experiments. The associated metabolites, structural properties, surface charges, and antibacterial activity of the AgNPs were also evaluated. Overall, a blue-shift of SPR peaks was observed for control AgNPs (λmax = 421 nm, 422 nm, 426 nm, and 406 nm for Cft-AgNPs, Cef-AgNPs, Cfx-AgNPs, and Cpm-AgNPs, respectively), compared to the control (λmax = 438 nm). Fourier-transform infrared spectroscopy showed that antibiotic-based AgNPs had distinct peaks that corresponded to the respective antibiotics, which were not observed in the control. XRD analysis showed that there were observed changes in crystallinity in antibiotic-based AgNPs compared to the control. TEM images revealed that all samples had spherical nanoparticles with different sizes and distributions compared to the control. The Zeta potential for extract-based AgNPs was − 33.6 mV, compared to -19.6 mV for Cft-AgNPs, -2 mV for Cef-AgNPs, -21.1 mV for Cfx-AgNPs, and − 24.2 mV for Cpm-AgNPs. The increase in the PDI value for antibiotic-based AgNPs also showed a highly polydisperse distribution. However, the antibiotic-AgNPs conjugates showed significantly higher activity against pathogenic bacteria. The addition of antibiotics to AgNPs brought significant changes in structural properties and antibacterial activities.

## Introduction

The World Health Organization (WHO) and United Nations have recognized antimicrobial resistance as a global public health crisis (Temkin et al. 2018). One of the public health concerns is the production of extended-spectrum β-lactamase (ESBL) enzymes in bacteria. These enzymes make the bacteria resistant to most β-lactam and cephalosporin antibiotics (Eftekhar et al. 2012). ESBLs are more frequently found in *Klebsiella pneumoniae*, an opportunistic bacteria known for causing infections in hospitalized patients, specifically those with a compromised immune system and other underlying diseases (Parveen et al. 2011). *K. pneumoniae* causes various human diseases, including pneumonia, bacteraemia, urinary tract infections, meningitis, pyogenic liver abscesses and sepsis. One of the looming problems has been the spread of *K. pneumoniae* strains resistant to cephalosporins due to less hospital hygiene and improper use of antibiotics (Wiener et al. 1999). ESBL-producing *K. pneumoniae* is associated with more than 55% mortality in case of such infections (Starzyk-Łuszcz et al. 2017). It has been estimated that *K. pneumoniae* strains have shown 49.02% resistance to Ceftazidime and variable resistance to other antibiotics along with Cephalosporins (Eftekhar et al. 2012). Similarly, other pathogens such as *Pseudomonas aeruginosa* are also known for their intrinsically advanced antibiotic resistance mechanisms. Studies have reported the presence of antibiotic-degrading enzymes such as extended-spectrum β-lactamases like AmpC cephalosporinases in bacteria including *Pseudomonas aeruginosa*, methicillin-resistant *Staphylococcus aureus*, and *Enterococcus faecalis* (Munita and Arias 2016).

These pathogens have been marked as priority pathogens for research and development of antibiotics against them (Temkin et al. 2018). However, owing to the over and improper use of antibiotics, the advanced mechanisms of antibiotic resistance in these pathogens require an alternative strategy to tackle them. Nanomaterials engineered to work against these bacterial strains are a workable alternative (Vazquez-Muñoz et al. 2019). For instance, silver nanoparticles (AgNPs) have been used as nano-antibiotics against Gram-positive and Gram-negative resistant bacterial strains (Panáček et al. 2009). Specific attributes of AgNPs, such as their generalized mode of action, antibacterial properties regardless of microbial susceptibility, and cost-effectiveness, promises benefit over conventional antibiotics (Vazquez-Muñoz et al. 2019). When synthesized via plant extracts, these AgNPs carry additional properties of being greener, environment-friendly, safer and biocompatible (Aromal and Philip 2012). Plant-based AgNPs have been used extensively against pathogenic bacteria. We have attempted to use extract from a potent antibacterial plant i.e. *Mentha longifolia* with low doses of existing cephalosporins for synthesizing AgNPs, which could possess properties of both extracts based AgNPs and antibiotics. *M. longifolia*, or wild mint, is one such plant from the family Lamiaceae valued for containing terpenoid oils which assist in reducing Ag into Ag° during AgNPs synthesis (Farzaei et al. 2017). This strategy is intended to decrease the requirement to provide a high dose of antibiotics and minimize nanoparticle cytotoxicity. Synthesizing nanoparticles in the presence of antibiotics not only enhances the antimicrobial activity of AgNPs, but also results in restoring the antibacterial efficacy against those resistant strains which had developed resistance against it. We, therefore, aimed to assess the combined effect of low doses of cephalosporins with the extract of *M. longifolia* on the synthesis, structural properties and antibacterial efficacy of AgNPs (Hassan et al. 2016).

## Methods

### Synthesis of silver nanoparticles

#### Plant material and extraction

The plant material was washed with the tap water, shade dried and ground into a fine powder. To extract metabolites, the plant powder was mixed with the distilled water at 1:40, respectively. The mixture was allowed to boil for 10 min on a hotplate at 100 °C. After cooling and precipitation of the residual plant material, the supernatant was collected and filtered using Whatman 2.5 μm filter paper to remove residues and debris. The filtrate was further cleaned from any residual particles through centrifugation (Javed et al. 2020).

#### Synthesis of antibiotic-supplemented silver nanoparticles

Silver nitrate solution (4 mM) and aqueous extract of *M. longifolia* were mixed in 1:2, respectively. Additionally, each of the antibiotics [Ceftazidime (Cft), Cefotaxime (Cef), Ceftriaxone (Cfx) and Cefepime (Cpm)] was added to separate reaction tubes along with an aqueous extract of *M. longifolia* and silver nitrate. The ratio of extract to AgNO_3_ to antibiotics in the reaction tube was kept at 1:2:1, respectively. The concentration of the stock solution for each antibiotic was 1 mg/mL. The reaction mixtures were kept under the shade on a normal sunny day for 3 h. The daylight intensity was measured to be approximately 20,000 lx. The apparent change in the colour of the reaction mixture from yellow to dark brown confirmed the reduction of the Ag + into Ag°. The reaction mixtures were subject to UV-Vis analysis for confirmation after three hours (Adil et al. 2019; Javed et al. 2020).

### Characterization of bio-synthesized silver nanoparticles

#### UV-Visible spectroscopy based confirmation of silver nanoparticles

To confirm the synthesis of AgNPs, the absorption bands of all the reaction mixtures were observed with a UV-visible spectrophotometer (Biobase BK-S360). The reaction mixture was diluted five fold in distilled water to obtain absorption peaks. Aqueous plant extract and antibiotic solutions were used as a control. An aliquot of 4 mL from each solution was taken in a crystalline Quartz cuvette and analyzed in a UV-visible spectrophotometer at 300–600 nm. After confirmation of AgNPs synthesis, the reaction mixtures were subject to Centrifugation two times at 13,000 rpm for 10 min to extract the AgNPs from the colloidal mixture. The obtained pellet was dried and kept for further analysis (Adil et al. 2019).

#### Transmission Electron Microscopy for size and morphology analysis

The size and distribution of AgNPs were compared to antibiotic-supplemented AgNPs through the analysis of the samples via Transmission Electron Microscope (TEM) (JEOL, JEM-2100, Japan). The sample images were taken at magnification up to 20 nanometres (nm). A single drop with a volume of 8 µL of the various nanoparticle suspensions was dried onto 200 mesh TEM grids with a carbon support film. Transmitted electron images were collected in bright field mode at an accelerating voltage of 30 kV with the specimen set at an 8 mm working distance. All the images were analyzed through Image J software for size variation in plant and antibiotic supplemented AgNPs. Similarly, the images were also analyzed for changes in the structure of AgNPs synthesized through supplementation with antibiotics.

#### X-Ray diffraction analysis

For analysis of the effect of antibiotics on crystal structure, X-ray diffraction (XRD) studies were carried out (X-ray diffractometer; JDX-3532, JEOL, Japan). The high-resolution XRD patterns were measured at 3 kW with a Cu target using a scintillation counter (λ = 1.5418 °A) at 40 kV and 40 mA and were recorded in the range of 2θ = 5°–80°.

#### Fourier transform infrared spectroscopy analysis

The presence of possible functional groups and biomolecules responsible for participating in the synthesis of AgNPs was confirmed using Fourier transform infrared (FTIR) spectroscopy. The AgNPs were resuspended in distilled water and were subjected to analysis in the range of 4000 − 400 waves cm^− 1^ via FTIR Spectrometer (Spectrum Two, Universal ATR, L1600235). The FTIR spectra of antibiotic-supplemented AgNPs were carefully analyzed for variations in absorption peaks compared to the controls, i.e., Extract of *M. longifolia*, extract-based AgNPs and antibiotic solutions.

#### Dynamic light scattering and Zeta potential measurement

The Dynamic light scattering (DLS) and Zeta potential analysis of AgNPs and antibiotic supplemented AgNPs were performed to measure their size and stability in suspension, respectively. The polydispersity index (PDI) was determined for nanoparticles to indicate their uniformity of formation and stability. A 3-gm dried extract based-AgNPs and antibiotic-supplemented-AgNPs were taken in 5 mL distilled water. The samples were then subjected to sonication (Power-Sonic 405) for 30 min. The hydrodynamic diameter and zeta potential of the samples were analyzed using Zeta-Sizer Nano (Nano-ZS ZEN 3600, Malvern) at 25℃ at a neutral pH.

#### Activity of silver nanoparticles against pathogenic strains

The antibacterial activity of the synthesized AgNPs was assessed by performing the Kirby–Bauer Disk Diffusion Susceptibility Test method (Bauer et al. 1966) against the four resistant pathogenic bacterial strains: *Pseudomonas aeruginosa* (ATCC 25,619), *Klebsiella pneumoniae* (ATCC 43,816), Methicillin-resistant *Staphylococcus aureus*, and *Enterococcus faecalis* (ATCC 29,212). The strains were spread using sterile cotton swabs on nutrient agar after overnight growth in liquid broth. Extract mediated AgNPs, and antibiotic-mediated AgNPs (Cft-, Cef-, Cfx- and Cpm-AgNPs), were applied to sterile disks in the same amount (5 µL each). The zones of inhibition were measured and represented in millimetres after incubating the plates at 37 ℃ for 24 h.

#### Haemolytic assays

To evaluate the cytotoxic effects of AgNPs, an in vitro haemolytic test was performed on blood collected from a healthy volunteer. A 3 mL volume of blood was added to anticoagulant citric acid tubes. Centrifugation was performed at 1500 x g for 5 min. The supernatant was discarded, and the pellet was washed three times using normal saline. The RBC pellet was suspended in a phosphate buffer reagent (pH 7.4). Various concentrations of the AgNPs i.e. (1.25, 2.5, 5, 10, 20 µg/mL) were applied to the suspended RBC. 1% Triton X-100 was used as a positive control, while phosphate buffer was kept as a negative control. The tubes were incubated at 37 °C for 2 h. After incubation, the samples were centrifuged again at 1500 x g for 10 min. After successful centrifugation, 100 µL of the supernatant was carefully added to a 96 well plate (Nelsonjoseph et al. 2022). Finally, the optical density was recorded at 490 nm using a microplate reader (TSx800 Absorbance Reader, BioTek Inc., USA). The percentage of hemolysis was calculated as:

$${\rm{\% }}\,{\rm{hemolysis}}\,{\rm{ = }}\,\left( {{\rm{SA - NA}}} \right){\rm{/}}\left( {{\rm{PA - NA}}} \right)\,{\rm{ \times 100}}$$

* SA = Absorbance of the sample.

* NA = Absorbance of the negative control.

* PA = Absorbance of the positive control.

## Results

### Characteristic comparison of antibiotic-supplemented silver nanoparticles

#### UV-Visible spectroscopy

**Fig. 1:**
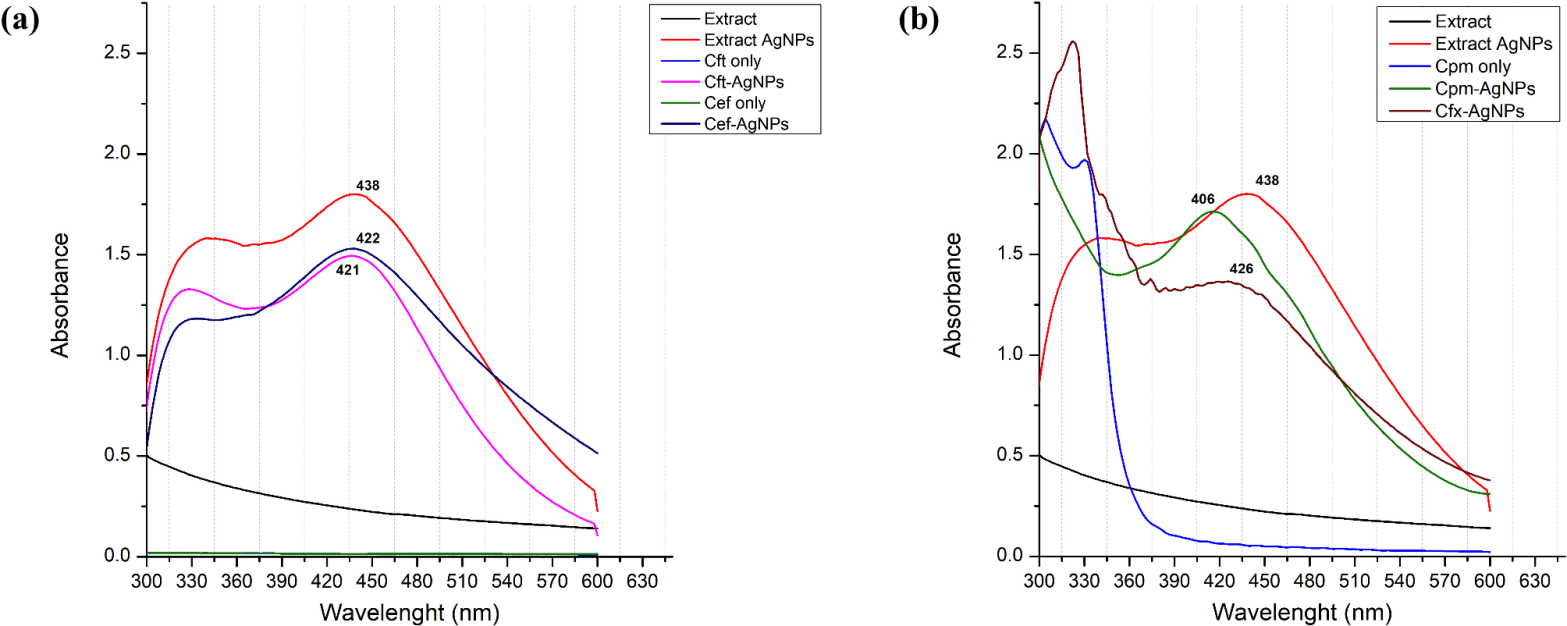
Comparative UV-VIS spectrums of *Mentha longifolia* extract based AgNPs, Cft-AgNPs and Cef-AgNPs (**a**) and. Cfx-AgNPs and Cpm-AgNPs (**b**). Plant extract and the respective antibiotics were used as control

AgNPs are synthesized because of the reduction of silver salt in the reaction mixtures by plant compounds. An apparent change in colour of the solution towards dark brown indicated the synthesis of AgNPs. The reduction of silver ions (Ag+) to AgNPs was confirmed by measuring the UV-Vis absorption of the reaction mixtures. Before performing UV-vis analysis, the sample was diluted five folds in distilled water while keeping distilled water as a baseline for reference. The AgNPs absorbed radiation between the range of 300–600 nm. In the case of plant extract based AgNPs, the maximum absorption (λ_max_) was recorded at 438 nm (Fig. 1a). Compared to extract-based AgNPs, the λ_max_ was 421 nm, 422 nm, 426 nm, and 406 nm for Cft-, Cef-, Cfx- and Cpm-AgNPs, respectively (Fig. 1a and b). Similarly, the absorbance of light in this range was higher in the case of AgNPs, while adding antibiotics reduced the absorption bands in this range (Fig. 1b).

#### Identification of functional groups involved in silver nanoparticles synthesis

**Fig. 2:**
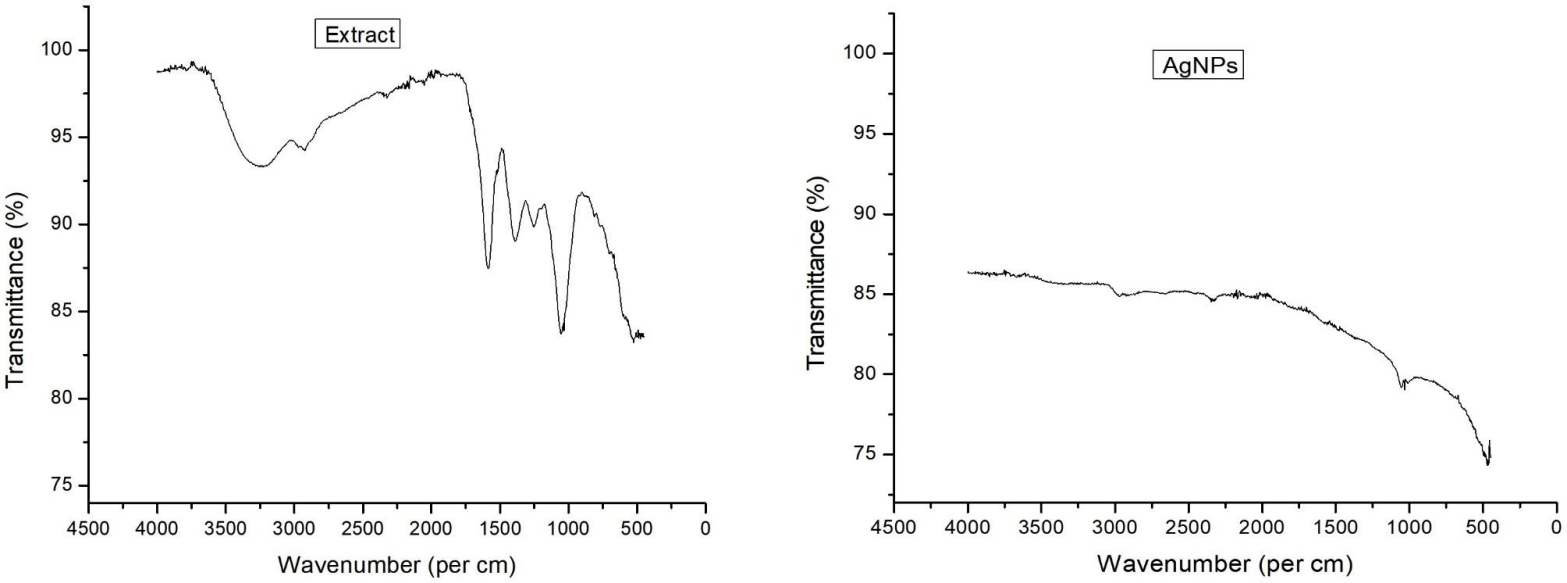
FTIR spectra of *Mentha longifolia* based AgNPs in comparison with the extract. The peaks correspond to functional groups possibly involved in the AgNPs capping and synthesis

FTIR analysis was performed to confirm the presence of potential surface functional groups responsible for differences in AgNPs and antibiotic supplemented AgNPs. The FTIR spectra of *M. longifolia* extract showed major peaks at 3264 cm^-1^, 2985 cm^-1^, 1587 cm^-1^,1386 cm^-1^, 1252 cm^-1^, 1051 cm^-1^, and 1033 cm^-1^ (Fig. 2). The broad peak from 3500 cm^-1^ to 3000 cm^-1^ corresponds to stretching vibrations due to -OH and -NH_2_ groups found in terpenoids, and flavonoids present in the extract of *M. longifolia*. The peak from 2800 cm^-to 1^ to 3000 cm^-1^ indicates the involvement of strong C-H bonds. The peak at 1386 cm-1 indicates the O–H bending vibrations of phenols, while the peaks at 1252 cm^-1^, 1051 cm^-1^, and 1033 cm^-1^ present the C–O stretching vibration of alcohols and ether present in the aqueous extract of *M. longifolia*. The FTIR spectra of plant extract based AgNPs showed distinct peaks at 2971 cm^-1^, 2333 cm^-1^, 1061 cm-1, 465 cm^-1^. The peak at 2971 cm-1 corresponds to the stretching vibrations of the C-H bonds of aldehydes. The peak at 2333 cm^-1^ could be assigned to the C ≡ C and C–N functional groups. These functional groups are due to carbonyl groups, aromatic rings, and primary and secondary amides of proteins found in the plant extract. These functional groups might also be linked with the terpenoids and phenols of plant extract. The peaks at 1061 cm^-1^ and 465 cm^-1^ correspond to -C-O vibrations of alcohols and C-H vibrations of aromatic rings (Fig. 2).

**Fig. 3:**
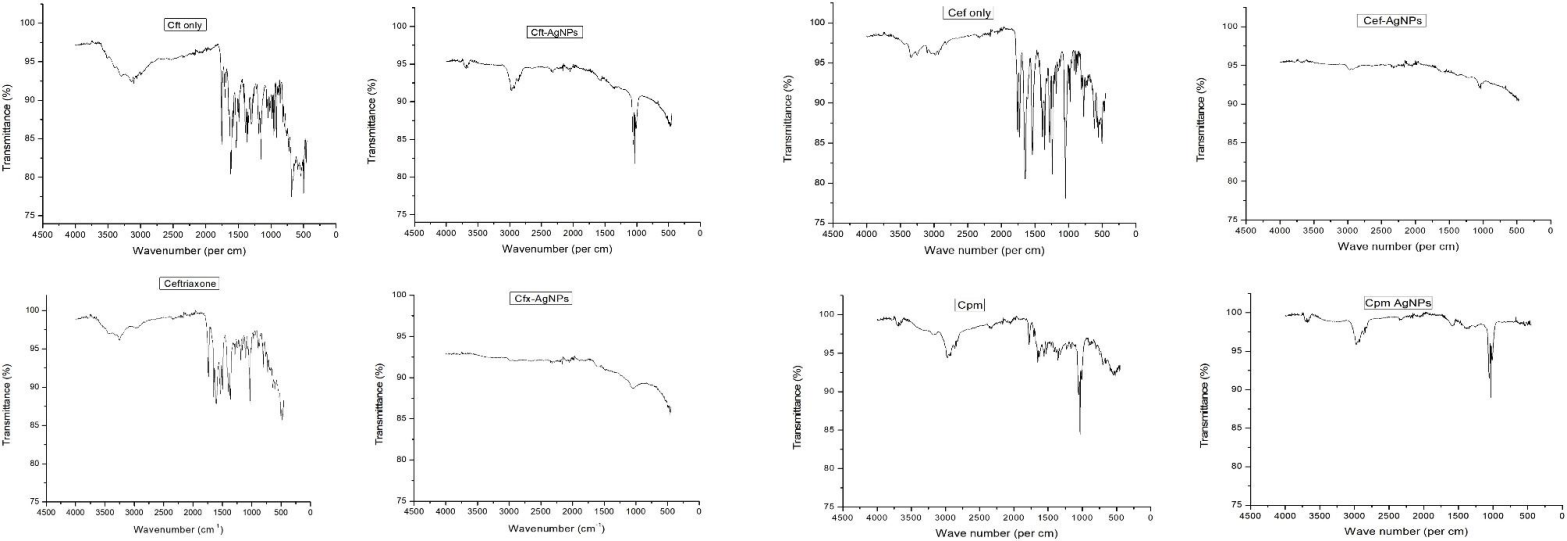
FTIR spectra of Cephalosporin mediated green synthesized AgNPs. The peaks correspond to functional groups possibly involved in the AgNPs capping and synthesis

The major peaks observed for Ceftazidime alone were 1765 cm^-1^, 1616 cm^-1^, 1534 cm^-1^, 1396 cm^-1^, 1157 cm^-1^ and 686 cm^-1^ (Fig. 3). The peak at 1765 cm^-1^ corresponds to the C = O stretching of carboxylic acid groups of the β-lactam ring. The peaks from 1300 to 1700 cm^-1^ could be attributed to the C = O carbonyl group and C-N of amides. The peaks at 1157 cm^-1^ and 686 cm^-1^ correspond to the stretching of C-O-C and -C ≡ C-H, respectively. The major peaks recorded for Cft-AgNPs were 3682 cm^-1^, 2973 cm^-1^, 2924 cm^-1^, 2846 cm^-1^, 2325 cm^-1^, 2084 cm^-1^, 1561 cm^-1^, 1034 cm^-1^, and 460 cm^-1^. After the synthesis of AgNPs, a significant reduction of bands was observed in almost all the corresponding peaks, which revealed the involvement of phenols and terpenoids in the synthesis and stabilization process. The band reduction at 1765 cm-1 (C = O), 1616 cm^-1^ (carbonyl group), 1396 cm^-1^ (C-N), 1157 cm^-1^ (C-O-C), and 686 cm^-1^ (-C ≡ C-H) confirmed the conjugation of Ceftazidime with AgNPs (Fig. 3).

For Cefotaxime alone, the FTIR spectra presented distinct peaks at 3328 cm^-1^, 1392 cm^-1^, 1350 cm^-1^, 1288 cm^-1^, 1239 cm^-1^, 1180 cm^-1^, and 1048 cm^-1^. The peaks at 3328 cm^-1^ indicate the O–H stretching vibrations of phenols and alcohols. The peak at 1392 cm^-1^ indicates the symmetric bending of C–H. The peaks at 1350 cm^-1^ and 1288 cm^-1^ indicate the C–N stretching of aromatic amines, and the peak at 1239 cm^-1^ indicates aryl -O stretch of Aromatic ethers. The peak at 1180 cm^-1^ indicates the C–N stretch of the amine group, while the peak at 1048 cm^-1^ indicates the strong C–O stretch of alcohols and carboxylic acid. For Cef-AgNPs, the FTIR spectra revealed major peaks at 2988 cm^-1^, 2930 cm^-1^, 2165 cm^-1^, 1060 cm^-1^, and 1032 cm^-1^. The peaks at 2988 cm^-1^ and 2930 cm^-1^ indicate the methyl group’s C–H stretching vibration. The peak at 2165 cm^-1^ indicates the C ≡ C stretching vibrations of terminal alkynes. Peaks at 1060 cm^-1^ indicate strong C–O stretch of alcohols, while the peak at 1032 cm^-1^ indicates Cyclohexane ring vibrations of the methylene group. The obtained FTIR spectra of Cef-AgNPs showed a reduction in C–H, C–N, and C–O bands compared to the spectra of pure Cefotaxime. These results suggest the efficient loading of Cefotaxime to AgNPs (Fig. 3).

The FTIR spectra of Ceftriaxone alone presented sharp peaks at 1738 cm^-1^, 1648 cm^-1^, 1607 cm^-1^, 1367 cm^-1^, 1032 cm^-1^, 805 cm^-1^, 483 cm^-1^, and a small peak at 3254 cm^-1^. The peak at 1738 cm^-1^ corresponds to the stretching vibration of 𝐶=𝑂 carbonyl of β-lactam, while the peak at 1648 cm^−1^ corresponds to the stretching vibration of 𝐶=𝑂 of carbonyl of amide. The absorption peak at 1607 cm–1 and 1367 cm^−1^ correspond to the stretching vibration of the carbonyl group and C-N, respectively. The peaks at 1032 cm^−1^ and 805 cm^−1^ could be assigned to C-O and C-H vibrations. The absorption band at 3254 cm^−1^ corresponds to the stretching vibration of the –OH group. After the synthesis of AgNPs, significant reduction of bands at 3264 cm^−1^, 1051 cm^−1^, 1032 cm^−1^, and 1033 cm^−1^ were recorded, which indicates the possible involvement of the C-O groups of alcohols in the synthesis and stabilization of Cfx-AgNPs. The band reduction at C = O 1738 cm^−1^, C = O 1648 cm^−1^, carbonyl group 1607 cm^−1^, C-N 1367 cm^−1^, and C-O 1033 cm^−1^ confirms the conjugation of Ceftriaxone with AgNPs (Fig. 3). For Cefepime and Cpm-AgNPs, the three major common peaks were observed at 3700 − 3640 cm^−1^, the second medium absorption peak at 3000 − 2890 cm^−1^, and the third intense absorption peak observed at 1100 − 1000 cm^−1^, 1080 − 1000 cm^−1^, and 1090 − 1000 cm^−1^ respectively. These peaks correspond to the strong O-H stretching vibrations of alcohol and C-O stretching vibrations of ester (Fig. 3). Finally, the FTIR spectra of Cefepime showed an additional peak at 2150 − 1880 cm^−1,^ which is also observed in Cpm-AgNPs with a slight shift at 2150 − 1995 cm^−1^ corresponding to C = C of terminal alkynes. After the synthesis of AgNPs, significant reduction of bands at 3264 cm^−1^, 1051 cm^−1^, 1032 cm^−1^, and 1033 cm^−1^ were recorded, which indicates the possible involvement of the C-O groups of alcohols in the synthesis and stabilization of AgNPs. Compared to the antibiotics alone, the distinguishing peaks altered (Reduction, broadening, disappearance, and/or frequency shifts) in antibiotic supplemented AgNPs could be linked with the conjugation of respective antibiotics with the nanoparticles. The corresponding IR spectra of all samples analyzed have been shown in Fig. 2 and Fig. 3.

#### Determination of the Zeta potential of antibiotic supplemented silver nanoparticles

**Fig. 4:**
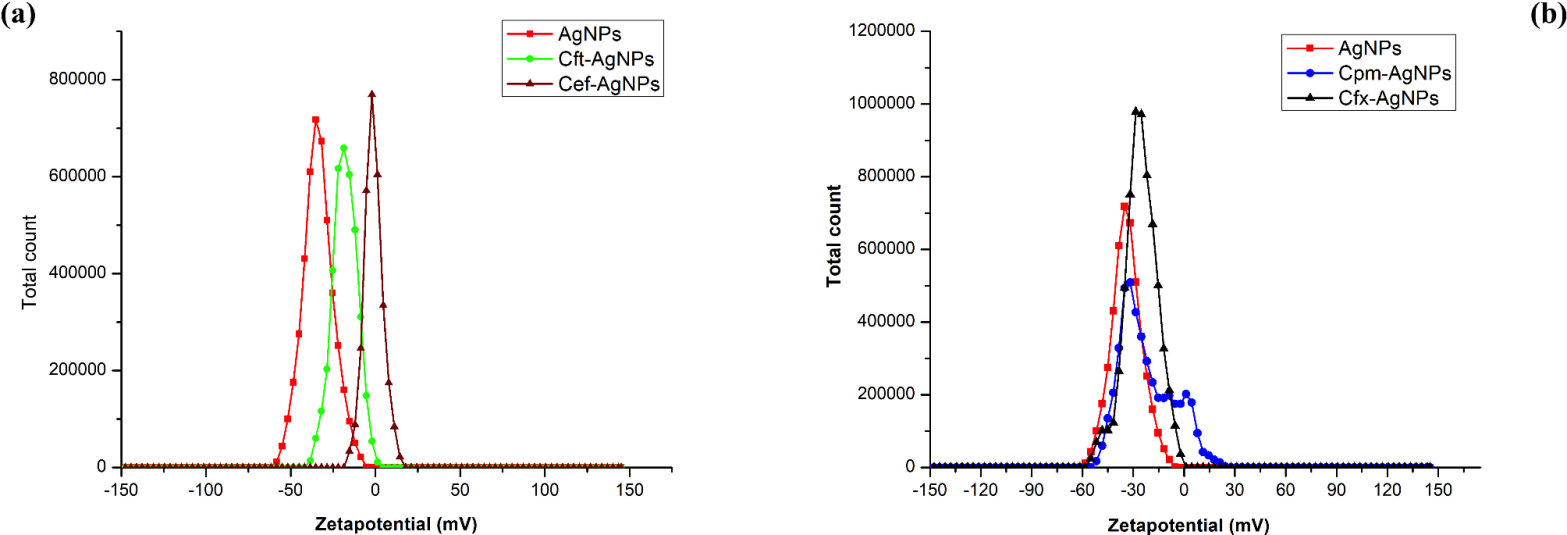
Comparison of zeta potential of extract-based AgNPs, Cft-AgNPs and Cef-AgNPs (**a**) and Cfx-AgNPs and Cpm-AgNPs (**b**)

The zeta potential of AgNPs and antibiotic supplemented AgNPs was measured to evaluate their suspension stability. The results revealed information regarding the stability and surface charge of the synthesized AgNPs. For extract based AgNPs, the average zeta potential value was − 33.6 mV. The results showed that zeta potential values for all antibiotic-supplemented AgNPs were lesser than plant-based AgNPs. The average zeta potential values were recorded as; -19.6 mV for Cft-AgNPs, -2 mV for Cef-AgNPs, -21.1 mV for Cfx-AgNPs, and − 24.2 mV for Cpm-AgNPs (Fig. 4a and b).

#### Determination of size of nanoparticles

The hydrodynamic diameter of both extract-based AgNPs and antibiotic-supplemented AgNPs was observed using dynamic light scattering (DLS). The hydrodynamic diameter recorded for extract-based AgNPs was 58.66 d.nm while 124.4 d.nm for Cft-AgNPs, 91.75 d.nm for Cef-AgNPs, 165 d.nm for Cfx-AgNPs, and 119.3 d.nm for Cpm-AgNPs. Similarly, the polydispersity index (PDI) was calculated as 0.333 for extract-based AgNPs and 0.430, 0.435, 0.727, 1.000, respectively for Cft-AgNPs, Cef-AgNPs, Cfx-AgNPs, and Cpm-AgNPs. The average zeta size was 18.2 nm for Cft-AgNPs, 15.7 nm for Cef-AgNPs, 13.5 nm for Cfx-AgNPs and 105.7 nm for Cpm-AgNPs.

#### Transmission Electron Microscopy of silver nanoparticles in comparison with antibiotic-supplemented silver nanoparticles

**Fig. 5:**
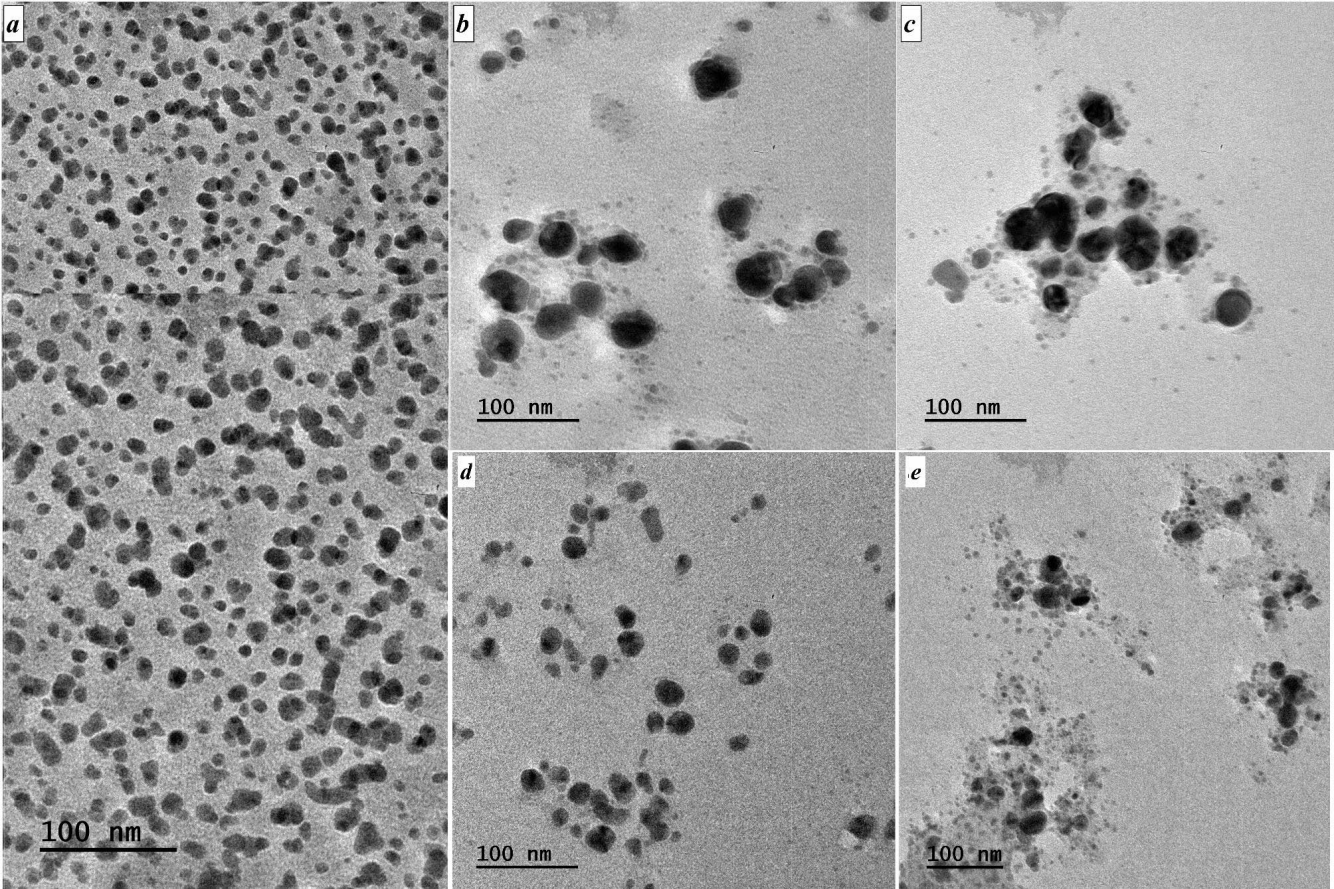
TEM images of (**a**) AgNPs, (**b**) Cft-AgNPs, (**c**) Cef-AgNPs, (**d**) Cfx-AgNPs and (**e**) Cpm-AgNPs

The shape and size of the nanoparticles were studied using TEM. Antibiotic-supplemented AgNPs were found to be more dispersed in sizes compared to AgNPs. The images showed that extract-based AgNPs were numerous and much smaller in size compared to the antibiotic-supplemented nanoparticles (Fig. 5). Among the extract based AgNPs, most of the nanoparticles were in the range of 4 to 23 nm averaging around 15 nm. The general shape of all the nanoparticles was spherical. For Cft-AgNPs, most nanoparticles were in the range of 9 to 48 nm in diameter with an average size of about 24 nm. The average size for Cef-AgNPs was 24 nm, with particles ranging from 8 to 50 nm.

Similarly, TEM images of Cfx-AgNPs revealed the average size to be 20 nm, with the smallest particle around 7 nm and the largest around 31 nm. Finally, the average particle size in the case of Cpm-AgNPs was recorded as 18 nm, with a size ranging from 7 to 28 nm. A closer high-resolution analysis of TEM images of all the antibiotic supplemented AgNPs showed structural changes around each particle, representing the accumulation of chemical groups around the particles (Fig. 5).

#### X-Ray diffraction analysis -based comparison of silver nanoparticles and antibiotic-supplemented silver nanoparticles

**Fig. 6:**
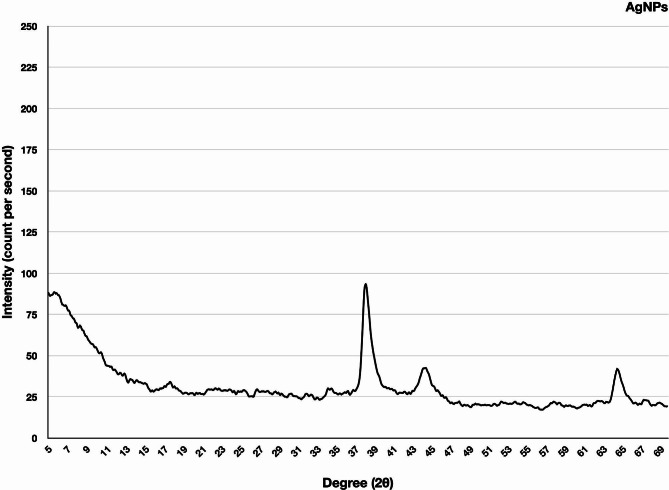
X-ray diffraction analysis of silver nanoparticles synthesized via extracts of *Mentha longifolia*

XRD was performed to determine the effect of antibiotics on the crystallinity of AgNPs. The XRD graph of extract mediated AgNPs indicated distinctive peaks at 2θ values of 38.4, 44.8, and 64.9 degrees which can be indexed to the (111), (200), and (220) reflection planes (Fig. 6). These values hint toward the typical pattern of AgNPs, which are found to have a face-centred cubic (fcc) structure related to the JCPDS standard 04-0783 (Kohan Baghkheirati et al. 2016). Furthermore, the Bragg peaks characteristic of Ag nanocrystals were also detected.

**Fig. 7:**
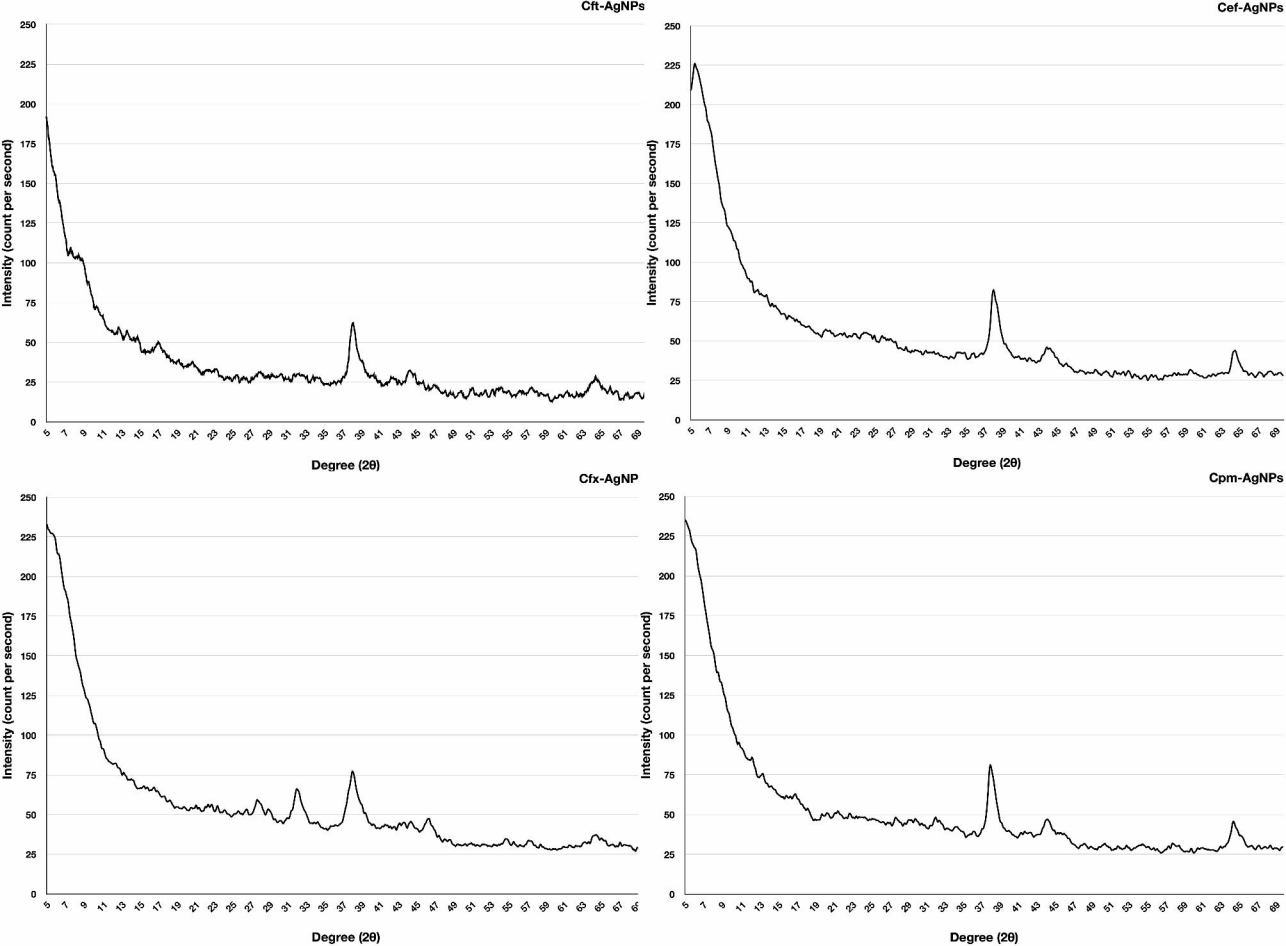
X-ray diffraction analysis of antibiotic mediated silver nanoparticles, including Cft-AgNPs, Cef-AgNPs, Cfx-AgNPs, and Cpm-AgNPs

The XRD graph of Cft-AgNPs showed distinct peaks at 2θ values of 38.15, 44.85, and 64.85. For Cef-AgNPs, the diffractogram presented distinct peaks at Bragg’s angles 2θ values of 38.35, 44.65, and 64.8 degrees. In the case of Cfx-AgNPs, the XRD graph indicated distinctive diffraction peaks at 2θ values of 27.7, 32.1, and 38.1 degrees. For Cpm-AgNPs, the XRD graph showed distinct diffraction peaks at 2θ values of 38.35, 44.7, and 64.8. All these diffraction peaks in antibiotic supplemented AgNPs can be related to the (111), (200), and (220) reflection planes of the fcc structure of nanosilver (Fig. 7). The sharp and narrow diffraction peaks revealed the nanoparticles’ extremely crystalline structure. The interaction between antibiotics and AgNPs could explain the small shift in the characteristic planes compared to the standard. The appearance of no additional peaks indicates the high purity of the nanoparticles.

### Antibacterial activity

**Fig. 8:**
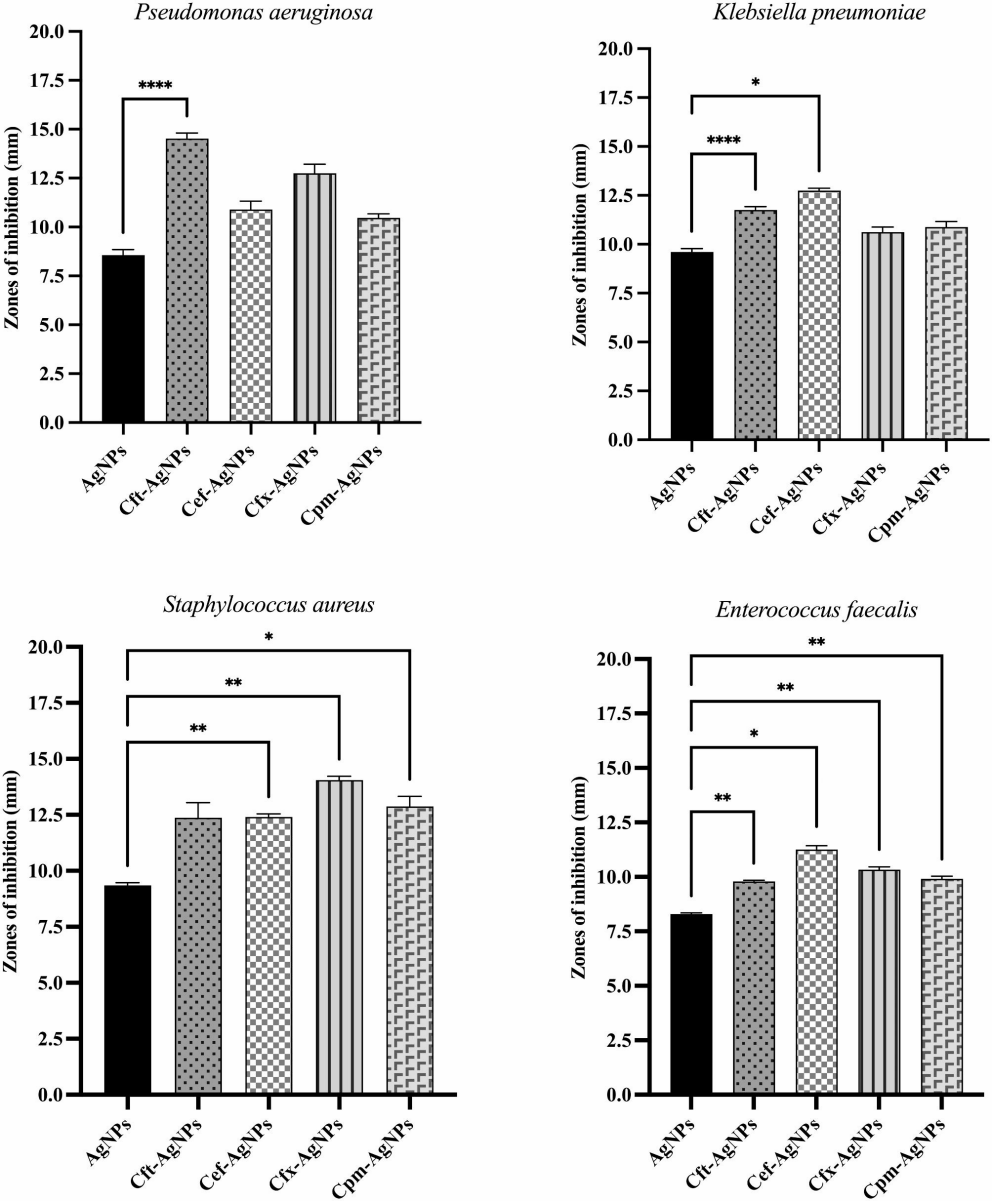
Results of antibacterial activity of plant based AgNPs, Cft-AgNPs, Cef-AgNPs, Cfx-AgNPs and Cpm-AgNPs against various resistant pathogens through disc diffusion method

The antibacterial activity of extract- and antibiotic-supplemented AgNPs was tested against four resistant pathogenic bacterial strains: *P. aeruginosa*, *K. pneumoniae*, Methicillin-resistant *S. aureus*, and *E. faecalis*. Compared to control, i.e., zones in AgNPs only (8.57 mm), the inhibition zones recorded against *P. aeruginosa* were 14.52, 10.90, 12.75, and 10.47 mm for Cft-AgNPs, Cef-AgNPs, Cfx-AgNPs and Cpm-AgNPs, respectively. Similarly, when applied against *K. pneumoniae*, Cft-AgNPs produced 11.75 mm zones, while a zone of 12.75 mm was recorded for Cef-AgNPs, 10.63 mm for Cfx-AgNPs and 10.88 mm for Cpm-AgNPs as compared to control (9.61 mm). The zones of inhibition recorded against MRSA were 12.37 mm, 12.40 mm, 14.05 mm and 12.88 mm for Cft-AgNPs, Cef-AgNPs, Cfx-AgNPs and Cpm-AgNPs, respectively, as compared to 9.33 mm in AgNPs. Finally, against *E. faecalis* the zones of inhibition measured 9.79 mm, 11.25 mm, 10.33 mm and 9.910 mm for Cft-AgNPs, Cef-AgNPs, Cfx-AgNPs and Cpm-AgNPs, respectively compared to 8.29 mm in control. Most of these zones were recorded to be significantly different from AgNPs alone (P < 0.05). The differences in the antimicrobial properties might have been due to the synergism of antibiotics and AgNPs. The conjugates of AgNPs and antibiotics produced enhanced antimicrobial potential as compared to AgNPs and antibiotics alone. Because, in conjugated form, the AgNPs core is surrounded by the supplemented antibiotics which provides increased concentration at the desired site and resulting in enhanced antimicrobial activities. The star represents significant values for Tukey’s multiple comparison test (Fig. 8).

#### Haemolytic assays for assessing the cytotoxicity of antibiotic-supplemented nanoparticles

The haemolytic effect of the plant extract mediated AgNPs and antibiotic-supplemented AgNPs was investigated against human erythrocytes. The results showed that the haemolytic action on the red blood cells for all types of nanoparticles was less than 5%. The highest haemolysis recorded was 3.09% in the case of Cef-AgNPs when administered against RBCs at a concentration of 20 µg/mL. The other antibiotic-supplemented AgNPs i.e., Cft-AgNPs, Cfx-AgNPs and Cpm-AgNPs showed 2.93%, 2.43%, and 2.22% haemolysis when applied in concentration of 20 µg/mL (Table 1). Compared to antibiotic-supplemented AgNPs, plant extract-based AgNPs showed 2.60% haemolysis while the positive control, i.e., Triton X-100, exhibited 100%, and the negative control, i.e., PBS buffer, exhibited 0.002% haemolysis. At lower concentrations, all types of AgNPs showed lesser haemolytic properties. The observed haemolytic profile of these AgNPs could be attributed to their surface chemistry, size, and physiochemical properties.

**Table 1:** Percent inhibition of human erythrocytes to determine the hemolytic potential of plant vs. antibiotic mediated AgNPs.

Nanoparticle type	Percent hemolysis (%) at different concentrations of nanoparticles (µg/mL)				
	20	10	5	2.5	1.25
AgNPs	2.609	1.436	0.928	0.175	0.501
Cft-AgNPs	2.931	1.851	1.515	1.021	0.671
Cef-AgNPs	3.093	1.903	1.797	1.092	0.921
Cfx-AgNPs	2.433	0.752	0.351	0.526	0.512
Cpm-AgNPs	2.224	1.427	1.412	1.397	0.767

## Discussion

Medicinal plants have been utilized to synthesize nanomaterials in the last few years. *Mentha longifolia* is also one of the medicinal plants from the family Lamiaceae. Many of these oil-producing plants are from the same family (Mashwani et al. 2016). The plant is known to contain the biomolecules in essential oils that are considered crucial agents in AgNPs synthesis (Song and Kim 2009). This study used the extracellular approach of synthesizing nanoparticles from *M. longifolia* aqueous extract. The apparent change in the colour of the reaction mixture was an indication of AgNPs synthesis, which was further confirmed by UV-Visible spectroscopy. A peak present in the range between 400 and 500 is generally attributed to AgNPs and is well documented for nanoparticles with a size ranging from 1 to 100 nm (Henglein 1993). This study recorded the maximum wavelength (λ_max_) at 438 nm for plant extract-based AgNPs. The essential oils found in the plant extract, such as terpenoid (monoterpenes and sesquiterpenes), are commonly observed to reduce the metallic silver to silver nanoparticles (Javed and Nadhman 2020). Compared with antibiotic-supplemented AgNPs, a blue shift was observed towards, i.e., 421 nm, 422 nm, 426 nm, and 406 nm for Cft-AgNPs, Cef-AgNPs, Cfx-AgNPs and Cpm-AgNPs, respectively. The blue shift might have occurred due to the involvement of various functional groups from antibiotics in the reduction and capping process, as confirmed by the FTIR results. The characteristics and patterns of functional groups involved in the capping of nanoparticles were revealed via FTIR analysis. Comparing the Infrared spectrum of concerned antibiotics and *M. longifolia* with that of extract-based AgNPs and antibiotic-supplemented AgNPs showed that functional groups such as phenols and alcohol, and terpenes are present in all samples suggests that these functional groups might be responsible for metal ion bio-reduction and nanoparticles synthesis.

Rapid aggregation of nanoparticles, along with their conjugating functional groups, may also be the reason behind the blue shift in wavelengths (Perveen et al. 2018). Another reason for the shift in the peaks might be the polarity difference between the excited and ground state. This difference corresponds to the increase or decrease in the energy gap between the ground and excited states. As explained by Mie’s theory, spherical metal nanoparticles should have only one SPR band in their absorption spectra, while anisotropic nanoparticles could have two or more SPR bands, depending on their form (He et al. 2002). A single SPR peak was seen in our study for all the synthesized nanoparticles, i.e., plant extract-based AgNPs, and antibiotic-supplemented AgNPs, indicating that our AgNPs were spherical, which correlates with the TEM results.

TEM analysis was carried out for both plant extract-based AgNPs, and antibiotic-supplemented AgNPs. From TEM images, it can be observed that extract-based AgNPs were in the range of 4–23 nm. While antibiotic-supplemented AgNPs were in the range of 7–50 nm. The nanoparticles were found to be polydisperse and spherical. The TEM images (Fig. 5) clearly show the existence of nanoparticles surrounded by layers of coating materials compared to images from extract-based AgNPs (Raut Rajesh et al. 2009). As confirmed by FTIR, the capping could be assigned to the conjugation of antibiotic functional groups to AgNPs.

Studies have reported that the difference in size between the NPs size analysis by TEM and DLS measurements is because DLS calculates the hydrodynamic diameter of the NPs. In contrast, TEM measures the metallic core of the NPs (Poda et al. 2011). The zeta potential value in the range of 0 to ± 10, ±10 to 20, and ˃30 mV indicates highly unstable, stable and highly stable nanoparticles, respectively (Ardani et al. 2017). The zeta potential analysis revealed the negative charge of both extracts-based AgNPs and antibiotic-supplemented AgNPs. The average zeta potential decreased from − 33.6 mV to -19.6 mV in Cft-AgNPs, -2 mV in Cef-AgNPs, -21.1 mV in Cfx-AgNPs, and − 24.2 mV in Cpm-AgNPs which indicates the formation of moderately stable AgNPs upon conjugation of antibiotics with the AgNPs. The surface charge shifted towards positive in the case of antibiotic-supplemented AgNPs. Aggregation between AgNPs and antibiotics might have occurred due to the attraction of these positive-negative charges. However, the negative value of zeta potential supports long-term stability, better colloidal nature, and high dispersity of the nanoparticles. A lower PDI value indicates evenly sized particles, while a higher PDI value shows a broader range of particle sizes. The PDI for extract based AgNPs was calculated as 0.333, while 0.430, 0.435, 0.727, and 1.000 for Cft-AgNPs, Cef-AgNPs, Cfx-AgNPs, and Cpm-AgNPs, respectively. The increase in PDI value indicates that the nanosystem has highly polydisperse distribution and that antibiotics have played a significant role in their fabrication. From the results of DLS and zeta potential, it can be concluded that while conferring a significant effect on the structure and chemistry of the nanoparticles, antibiotics have decreased their colloidal stability. Further engineering of these nanomaterials will be needed to increase their stability in liquid systems.

The crystal structure of these nanoparticles was analyzed using XRD results. Figure 6 & 7 shows the XRD patterns of extract-mediated AgNPs and antibiotic-supplemented AgNPs. Extract-mediated AgNPs and antibiotic-supplemented AgNPs both crystallized to form the face-centred cubic structures. This result correlated well with the previous studies reporting plant extracts-based synthesis of AgNPs (Jeyaraj et al. 2013). In neither of the diffractograms, there was no evidence of any of the impurity or oxide phases. All the diffractograms include diffraction peak positions that are quite like the conventional diffraction patterns of Ag (JCPDS 04-0783). Diffractograms with broad peaks suggest that nanoparticle crystallite sizes are small (Khurana et al. 2016).

The development of rapid antimicrobial resistance is a serious threat towards human life. Once an individual is infected by a multi drug resistant pathogen, the infected person will need to be treated with broad spectrum antibiotics that are toxic as well as less effective (Webb et al. 2005). The researchers are trying to utilize metal nanoparticles especially AgNPs to overcome drug resistance **(**Gemmell et al. 2006). AgNPs itself are strong antimicrobials but they are toxic and not environment friendly as compared to green synthesized AgNPs. Green synthesized AgNPs possess lower cellular toxicity and are environment friendly. The green synthesized AgNPs when conjugated with low doses of antibiotics possesses higher antimicrobial potential as compared to AgNPs alone and antibiotics alone. The conjugated form of AgNPs have larger surface area which favours the enhanced antimicrobial activity (Thomas et al. 2020). Higher doses of AgNPs alone and antibiotics alone are required to achieve the same results as conjugates of AgNPs and antibiotics which contain smaller quantity of AgNPs and antibiotics. Briefly, the antibiotic-AgNPs conjugates reduces the antibiotic dose required to achieve similar results which makes it safe and much effective (Fayaz et al. 2010). It has been reported that when the nanoparticles are conjugated with antibiotics, the quantity of drug required is lower, which ultimately lowers its toxicity and minimizes the risk of resistance development (Zhou et al. 2016).

In this study, we analyzed the combined effects of plant extract and antibiotics while synthesizing AgNPs against pathogenic strains of bacteria, i.e., *P. aeruginosa, K. pneumoniae, S. aureus*, and *E. faecalis* using disc diffusion antibacterial assay. The reason behind the selection of these pathogens is their resistance to Cephalosporins. The inhibition zones in each type of AgNPs confirmed that the conjugates of AgNPs and antibiotics except Cpm-AgNPs possess higher antimicrobial potential than AgNPs and antibiotics alone while working synergistically. According to Dunnett’s multiple comparison test, the results in the case of Cft-AgNPs, Cef-AgNPs, and Cfx-AgNPs were significantly different from control, i.e., plant extract based AgNPs (p < 0.05). The enhanced effect of AgNPs with antibiotics for all the conjugates except Cpm-AgNPs was notably seen against all the tested pathogens. Although the results showed significant differences among zones of inhibition, the lack of drastic differences between them points toward one possibility. As can be seen with all antibiotic supplemented AgNPs, the lower zeta potential indicates their lesser stability once they get functional groups from antibiotics during synthesis. This means that AgNPs might form agglomerates and thus cannot travel farther in the gelled medium for disc diffusion compared to their potential against bacteria (Kourmouli et al. 2018).

The exact mechanism is still under investigation; however, studies have proposed that the conjugates of AgNPs and antibiotic complex will release Ag^+^ at a higher rate than AgNPs alone. Moreover, it has also been reported that the conjugation of AgNPs and antibiotics will effectively increase antibiotic concentration at the desired site (Allahverdiyev et al. 2011). Another study reported that bacteria’s cell wall is ruptured by Ag^+^ ions released from AgNPs, ultimately leading to cell death (Yu-sen et al. 1998). Another mechanism includes the combination of oxygen and silver and its reaction with sulfhydryl groups present on the cell wall, resulting in R–S–S–R bonds, thus inhibiting respiration and ultimately leading to cell death (Kumar et al. 2004). The tested bacteria, usually Cephalosporins resistant, were much more susceptible to AgNPs, especially Cft-AgNPs, Cef-AgNPs, and Cfx-AgNPs. Hence, the application of antibiotic-supplemented AgNPs is suggested as an alternative solution to the resistance developed by the pathogens against Cephalosporins (Harshiny et al. 2015). In addition, we also studied the cellular toxicity of AgNPs. We performed the haemolytic assay of both extract-based AgNPs and antibiotic-supplemented AgNPs, and the results correlated with the previous studies (Balaji et al. 2009). The results showed that the extract-based AgNPs and antibiotic-supplemented AgNPs did not cause any harm to RBCs in lower concentrations. This validates the utilization of biologically synthesized NPs compared to the NPs synthesized through physical and chemical methods that are more toxic.

AgNPs can be utilized with various antibiotics as a novel therapy for multidrug-resistant clinical isolates (Swami et al. 2019). By supplementing antibiotics during the synthesis of nanoparticles, the toxicity of both AgNPs and drugs towards human cells decreases, while their bactericidal efficacy against resistant bacteria increases (Hassan et al. 2016). AgNPs are selective toward cell membranes, allowing them to act as drug carriers by delivering antibiotics to the cell surface. AgNPs bind to the sulphur-containing proteins and increase the cell membrane’s permeability. As a result, antibiotic penetration into the cell is made easier. Combination approaches for preventing antibiotic resistance are receiving a lot of attention, and they’re used to administer low doses of antibiotics. As a result, pathogens’ chances of developing resistance are reduced (Adil et al. 2019). The results revealed the synergistic effect of AgNPs on all the tested pathogens in conjugation with Ceftazidime, Cefotaxime, and Ceftriaxone. The antibiotic-supplemented AgNPs showed increased antimicrobial efficacy as compared to AgNPs and antibiotics alone. Both AgNPs and antibiotic-supplemented AgNPs possess lower cellular toxicity to RBCs in lower concentrations. Thus, the prepared antibiotic-supplemented AgNPs would pave the way for effective treatment and could serve as alternative medicine against Cephalosporin-resistant pathogens. Further experimental studies are required to investigate the mechanism of synergism of antibiotic-supplemented AgNPs.

## Data Availability

Not Applicable.
